# Fetal membrane imaging: current and future perspectives—a review

**DOI:** 10.3389/fphys.2024.1330702

**Published:** 2024-06-27

**Authors:** Dan Wu, Jiasong Cao, Meiyi Xu, Cunling Zhang, Zhuo Wei, Wen Li, Ying Chang

**Affiliations:** ^1^ Tianjin Institute of Obstetrics and Gynecology, Tianjin Central Hospital of Obstetrics and Gynecology, Tianjin, China; ^2^ Tianjin Key Laboratory of Human Development and Reproductive Regulation, Tianjin, China; ^3^ Nankai University Affiliated Hospital of Obstetrics and Gynecology, Tianjin, China

**Keywords:** obstetrics, fetal membranes, premature birth, ultrasonography, prenatal diagnosis

## Abstract

Fetal membrane providing mechanical support and immune protection for the growing fetus until it ruptures during parturition. The abnormalities of fetal membrane (thickening, separation, etc.) are related to adverse perinatal outcomes such as premature delivery, fetal deformities and fetal death. As a noninvasive method, imaging methods play an important role in prenatal examination. In this paper, we comprehensively reviewed the manuscripts on fetal membrane imaging method and their potential role in predicting adverse perinatal fetal prognosis. We also discussed the prospect of artificial intelligence in fetal membrane imaging in the future.

## Introduction

The fetal membranes (FMs) is mainly composed of the amnion, chorion and decidua, which provide mechanical support and immune protection for the growing fetus until it ruptures during parturition. In recent years, although some studies have focused on the immune and physical properties of FMs, there are still few studies on FMs especially in image studies, which are usually considered “dead tissue” (Menon and Moore, 2020).

The main abnormalities of FMs are chorioamniotic separation (CMS), FMs thickening and hematoma. Normally, the chorion and amnion fuse together in middle pregnancy (14–16 weeks), while CMS after 16 weeks is considered abnormal, which is related to adverse perinatal outcomes such as premature delivery (PTB), fetal deformities, and fetal death (Qi et al., 2020). The inflammatory environment leads to the thickening of the FMs (Severi et al., 2008), this change in FMs may cause preterm premature rupture of membranes (pPROM), and leading to premature delivery (Menon and Fortunato, 2007). Hematomas of FMs are relatively rare, it associated with fetal distress and perinatal death (Richards and Bennett, 1998). In summary, healthy FMs are necessary for the healthy growth of the fetus and abnormal fetal membranes are closely related to poor perinatal clinical prognosis (Menon and Moore, 2020). The assessment of fetal membrane (FM) conditions during pregnancy holds crucial clinical significance.

Ultrasound remains the predominant imaging modality employed in obstetrics, extensively utilized to visualize fetal membranes and investigate the association between FM abnormalities and perinatal fetal clinical outcomes across a multitude of studies (Kaufman et al., 1985; Graf et al., 1997; Lewi et al., 2004; Severi et al., 2008; Schlehe et al., 2014; Gerson et al., 2019; Ghose et al., 2021). Magnetic resonance imaging (MRI) is also used to image the FMs, investigations in this domain remain relatively scarce (Qi et al., 2020). In addition to the imaging of FMs *in vivo,* researchers also used the optical coherence tomography (OCT) method to image FMs *in vitro* to obtain a complete biological structure of FMs (Ren et al., 2011; Micili et al., 2013; Avila et al., 2014).

FM imaging, such as ultrasound and MRI, uses noninvasive methods to visualize the structure of the FMs, which can predict the health of the fetus in the perinatal period with less danger to pregnant women and the fetus (Nunes et al., 2016), while *in vitro* imaging of the FM is of great significance to the study morphological details of human FMs (Ren et al., 2011). These imaging methods can provide meaningful information for the study of fetal membranes and the study of pPROM.

Regrettably, conventional imaging examinations often neglect the identification of abnormal conditions such as CMS and FM thickening. Moreover, existing ultrasound instruments lack automated capabilities for the detection and characterization of fetal membrane abnormalities. This circumstance may inadvertently lead clinicians to overlook crucial information pertaining to the prediction of preterm birth during ultrasound examinations. The advancement of machine learning has enabled computers to assist clinicians in diagnosing medical conditions by learning and analyzing features extracted from medical images. This approach holds potential for the recognition and identification of FM abnormalities as well.

**FIGURE 1:**
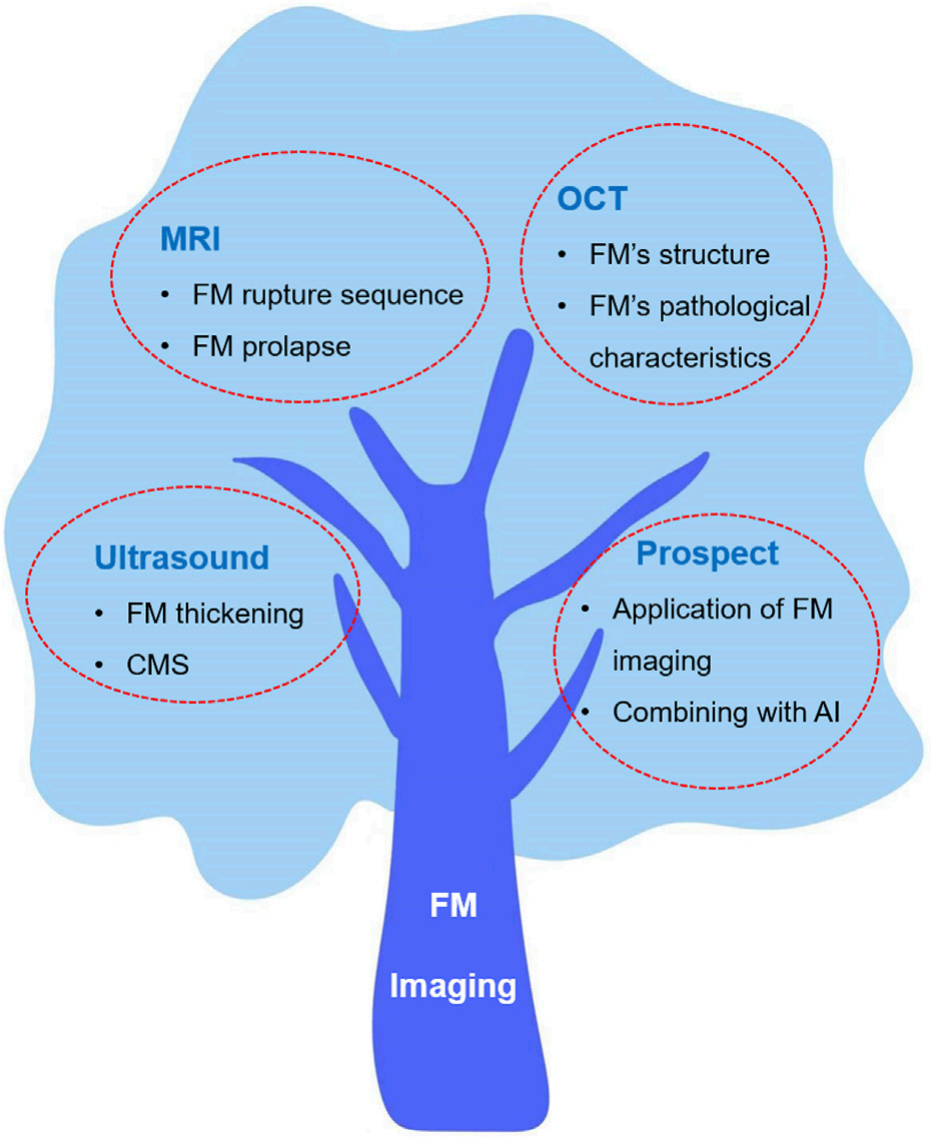
The overview of this review.

The purpose of this review is to summarize the latest research results of FM imaging and discuss the detection capabilities of various imaging technologies. We also discuss the relationship between abnormal FMs and adverse perinatal fetal clinical outcomes in these works, as well as the application and prospects of FM imaging in the future. These discussions may provide some reference opinions for the diagnosis of diseases related to fetal membrane abnormalities in the future. Finally, we have explored the prospective role of artificial intelligence in aiding the diagnosis of FM imaging, offering novel insights for the integration of medical practices with the industrial sector. This discussion endeavors to stimulate further considerations and potential avenues for future research and collaboration. Figure 1 shows the overview of this review.

## Materials and Methods

This review comprehensively searched the manuscripts on FM imaging and its potential role in predicting adverse perinatal fetal prognosis. We searched the PUBMED database for “amnion,” “chorion,” “fetal membrane,” “ultrasound,” “MRI” and “imaging” and reviewed the references in relevant articles. All the research comes from English publications and all studies involved FM imaging research, some studies involved fetal prognosis after FM imaging. When collecting literature, we excluded single case reports, and the sample size in each study was ≥10. This comprehensive review aims to consolidate and synthesize a wide range of *in vivo* and *in vitro* FM imaging studies, thereby enhancing the overall comprehensiveness of this review.

After determining the relevant papers, we extracted important research features to build a table. The relevant data include author, year of publication, *in vivo* or *in vitro* research, imaging method, sample size, observation objects, results and limitations. Table 1 summarizes all FM imaging studies *in vivo* or *in vitro.*

**TABLE 1:** Summary of fetal membrane imaging research.

Author	Year	*In Vivo* or *in vitro*	Method	Sample size	Observed object	Results	Limitations
Frigo et al. (1996)	1996	Both	Ultrasound and microscopy	28 normal deliveries	Thickness of FM	Membrane thickness correlation coefficient by ultrasound vs histology, *r* = 0.96, *p* < 0.01	Limited sample size, term deliveries only
Frigo et al. (1998)	1998	Both	Ultrasound and microscopy	32 between gestational weeks 28 and 32	Thickness of FM	Membrane thickness in pPROM group was thinner, *p* < 0.01	Limited sample size, lack of precise location of fetal membrane measurement
Severi et al. (2008)	2008	*In vivo*	Ultrasound	158 between gestational weeks 18 and 35	Thickness of FM	Women who delivered preterm had a greater membrane thickness than those who delivered at term, *p* < 0.01	lack of precise location of fetal membrane measurement, fetal membranes only measured once
Basaran et al. (2014)	2014	*In vivo*	Ultrasound	190 between gestational weeks 18 and 22	Thickness of FM	No difference in fetal membrane thickness in the second or third trimester between those who delivered term vs preterm	Low proportion of premature delivery
Kaufman et al. (1985)	1985	*In vivo*	Ultrasound	13(7 with separation of FM; 6 elevated chorion)	Separated chorioamnion and elevated Chorion	CMS is not associated with vaginal bleeding	Limited sample size, no statistical analysis
Gerson et al. (2019)	2018	*In vivo*	Ultrasound	23 patients with separation of FM	Separation of FM	Continuous separation of fetal membranes is closely related to adverse clinical outcomes of the fetus	No control group, no statistical analysis
Wilson et al. (2003)	2003	*In vivo*	Ultrasound	53 patients underwent fetal surgery	Separation of FM	The number of gestational weeks during operation is an important factor for fetal membrane separation (*p* < 0.05) and premature rupture of membranes (*p* < 0.01)	Prospective research is required
Soni et al. (2016)	2015	*In vivo*	Ultrasound	88 patients underwent fetal myelomeningocele repair	Separation of FM	Chorioamniotic membrane separation is significantly related to premature rupture of membranes (*p* = 0.08) and premature delivery (*p* = 0.01), The repair of fetal spinal cord meningocele was performed at <23 weeks of pregnancy, which was related to premature rupture of membranes and separation of membranes	Prospective research is reqired
Sydorak et al. (2002)	2022	*In vivo*	Ultrasound	75 patients underwent fetal surgery	Separation of FM	The incidence of premature rupture of membranes (63% vs. 45%), premature delivery (57% vs. 38%) and chorioamnionitis (29% vs. 15%) in CMS patients increased, but there was no difference in mortality	No statistical analysis
Qi et al. (2020)	2020	*In vivo*	MRI	18 between gestational weeks 20 and 36	Abnormal fetal membrane	The rupture sequence of fetal membranes is consistent with the model studied *in vitro*	Small sample size, lack of other characteristics observation (thickness, *etc.*)
Qi et al. (2022)	2022	*In vivo*	MRI	77 between gestational weeks 28 and 37	FM prolapsed depth >5 mm FM signal abnormalities	FM prolapse >5 mm and FM signal abnormalities, were associated with PROM (*p* < 0.01) and PPROM (*p* < 0.01)	Small sample size and potential selection bias
Ren et al. (2011)	2011	*In vitro*	OCT	10 FMs, 60 samples	Fetal membrane structure	OTC can divide the fetal membrane structure into four layers, could also detect the MCP of FMCT layer	The imaging depth of OTC is limited
Micili et al. (2013)	2012	*In vitro*	OCT	45 FMs	Thickness of FM	The fetal membrane thickness of NB group was the thickest, PRB group was the thinnest, while that of pPROM group was between NB and PRB groups	Few measurements
Avila et al. (2014)	2014	*In vitro*	OCT	10 FMs	Fetal membrane structure and pathological characteristics	OTC cleaning shows the structure of fetal membranes, including amnion, the space under amnion, chorionic reticular layer and chorionic trophoblast decidua. The amnion is shown as a bright band at the bottom of the OCT image. Other histopathological features identified by OCT include microscopic chorionic pseudocyst, ghost chorion, calcification, meconium staining, and chorioamnionitis	Small sample size, only morphology was observed, not related to clinical disease

## Result

### FMs in ultrasound

Noninvasive prenatal examinations methods are often the best choice for pregnant women because invasive inspection methods (amniocentesis, etc.) will not only cause pain to pregnant women but also cause adverse events such as CMS and miscarriage (Borlum, 1989; Barak et al., 2003). Consequently, imaging modalities assume a crucial role in facilitating prenatal examinations.

**FIGURE 2:**
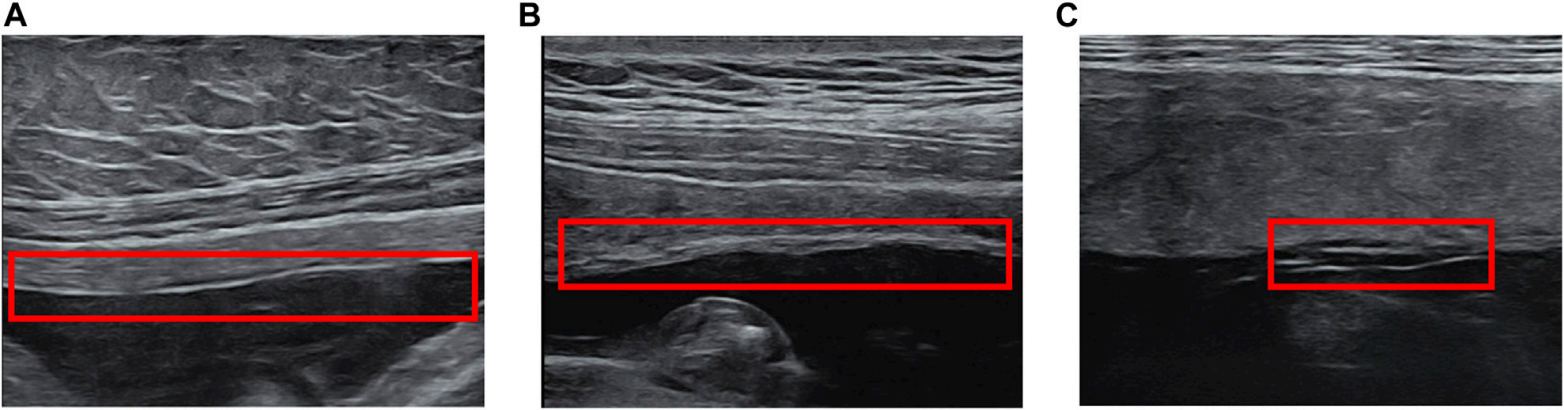
Fetal membrane abnormalities in ultrasound. **(A)** Normal FM **(B)** FM Thickening **(C)** Chorioamniotic Separation.

Many studies on FM imaging use ultrasound methods. These studies mainly focus on the thickness (Figure 2B) and separation of FMs (Figure 2C), which may be related to poor perinatal fetal prognosis such like pPROM and preterm birth (Qi et al., 2020). Many researchers are concerned about the correlation between FM’s thickness and premature delivery.

As early as 1996, Frigo et al. (1996) used ultrasound to measure FM’s thickness *in vivo* and compared it with the results measured by an optical microscope of FM tissue. They measured the FM thickness after 28 normal deliveries; the FM thickness measured by ultrasound was 0.83 mm ± 0.11 mm (0.72–1.08 mm), and the FM thickness measured by optical microscopy was 0.82 ± 0.13 (0.71–1.10 mm). The results showed that the *in vivo* ultrasound examination results were highly correlated with the light microscopy measurement results (*r* = 0.96, *P* < 0.01).

To extend their first study, Frigo et al. took the thickness of FM measured by ultrasound as an indicator to research the relationship between FM thickness and premature rupture of membranes (Frigo et al., 1998). In this study, they observed FMs in 32 patients between gestational weeks 28 and 32. Similar to their first study, the thickness of ultrasound and microscopy measurements were highly correlated (correlation coefficient *r* = 0.92, *P* < 0.01). The FM thickness of the pPROM group was 0.54 ± 0.9 mm, which was significantly different from that of the control group, with an FM thickness of 0.74 ± 1.01 mm (*P* < 0.01). The FM in the control group were thicker than those in the pPROM group.

Severi et al. performed ultrasound examinations on 158 pregnant women between 18 and 35 weeks of gestation. They used 2.5–6.6 MHz transabdominal probes to measure the FM area 3 cm from the umbilical cord. The average FM thickness of premature infants was 1.67 ± 0.27 mm, and the average FM thickness of full-term infants was 1.14 ± 0.3 mm (*P* < 0.01). The FM of the premature group was thicker. In addition, they found that there was a statistically significant negative correlation between the FM thickness and the gestational age at delivery (*r* = −0.302, *p* < 0.01), and it shows that the thicker the FMs, the smaller the gestational age at delivery. This study also shows that the thickness of the membrane near the cervix may indicate infection, but it is more suitable to use vaginal ultrasound for examination (Severi et al., 2008).

Basaran et al. (2014) conducted an ultrasound study of FMs, which examined the predictive effect of FM thickness on premature delivery in asymptomatic pregnant women. In this study, 190 pregnant women between 18 and 22 weeks of gestation were included, and the FM thickness was measured with a 5.7 MHz ultrasound probe in the second and third trimesters of pregnancy. The FM’s thickness in the second trimester of term delivery was 0.79 mm ± 0.23 mm, while that in the preterm delivery group was 0.77 mm ± 0.27 mm (*P* = 0.452). Additionally, in the third trimester, there was no significant difference in the thickness of FM. Between the term delivery group and the preterm delivery group (0.88 mm ± 0.27 and 0.91 ± 0.20 mm, *P* = 0.448), which is different from the results of previous studies (Frigo et al., 1996; Severi et al., 2008). The author believes the limitation in this study is artifacts in ultrasound images and different mechanisms of premature delivery (Basaran et al., 2014).

In addition to thickening of the fetal membrane, separation of the chorioamniotic membrane is also a common fetal membrane abnormality in ultrasound. Normally, in the first and second trimesters of pregnancy (14–16 weeks) (Gerson et al., 2019), the amnion and chorion fuse. After 16 weeks of pregnancy, any CMS is considered abnormal (Qi et al., 2020). Many studies and case reports indicate that CMS is related to adverse clinical outcomes, such as premature rupture of membranes and fetal extremity deformities (Frigo et al., 1996; Graf et al., 1997; Frigo et al., 1998; Wilson et al., 2003; Kim et al., 2007; Nunes et al., 2016; Soni et al., 2016; Ghose et al., 2021). In addition to spontaneous CMS, such separation can also occur after fetal surgery and amniocentesis (Sydorak et al., 2002; Soni et al., 2016). CMS could be found when there was a lucent space between the uterus and chorion, with a fine mobile linear echo (Figure 2C). The separated FM can surround the fetus and even extend to the fetal surface of the placenta (Burrows et al., 1982).

In 1985, Kaufman et al. (1985) used ultrasound to observe CMS. They divided the membranes into two groups, the CMS group and the elected chorion group. Among the seven pregnant women with CMS, five women were delivered at term and gave birth to healthy infants, the other two women had a premature delivery, one of them had placental chorioangioma, while the other patient’s stillborn fetus had a cystic hygroma indicative of Turner’s syndrome. Among the six patients with elevated chorion, vaginal bleeding occurred in the early and middle stages of pregnancy. Among them, five patients delivered healthy babies at term, and one patient aborted due to elevated chorion. The authors believe that CMS is usually not related to vaginal bleeding, but the elevated chorion is related to vaginal bleeding.

In 2018, Kristin D et al. published a study. They used the hospital’s ultrasound database to search for cases of CMS, and included 23 samples for review. Among these 23 cases, 13 obstetric complications (56.5%), 2 fetal deaths (8.7%), and 8 fetal malformations (37.8%) were observed. Of the 20 samples with placental records, 11 (55%) had poor placental perfusion. All these cases indicate that the continuous separation of the amnion and chorion was closely related to the adverse clinical outcome of the fetus such like premature delivery and fetal malformation (Gerson et al., 2019).

In addition to spontaneous CMS, fetal surgery is also an important cause of CMS and may lead to serious adverse clinical outcomes. In 2003, Wilson et al. (2003) used ultrasound to follow up the CMS after open fetal surgery and divided the samples into three groups according to the time of FM separation, namely, not present (NP), immediate (<2 weeks) and delayed (>2 weeks). The types of surgery included myelomeningocele (MMC); cystic adenomatous malformation (CCAM); congenital diaphragmatic hernia (CDH) and sacrococcygeal endometriosis (SCT). In this research, no membrane separation was observed in the CCAM, CDH and SCT groups. In the MMC group, 19 cases (44%) were operated on before 23 weeks, and 24 cases (56%) were operated on after 23 weeks of pregnancy, which indicated that the gestational week during operation is an important factor for fetal membrane separation (*P* < 0.05).

Shelly Soni et al. (2016) reviewed the ultrasound images of 88 patients who had undergone repair surgery for myelomeningocele. Among the 88 surgical patients, 27 (30.7%) had premature rupture of membranes, 21 (23.9%) had CMS, of which 10 (47.6%) had complete CMS, and 11 (52.4%) had local CMS. Fetal surgery at early gestational age is an important risk factor related to CMS (*P* = 0.01) and premature rupture of membranes (*P* < 0.01). Additionally, the CMS was significantly related to pPROM (59.1% vs. 21.2%, *P* = 0.008) and preterm birth (32.1 + 4.2 weeks vs. 34.3 + 3.5 weeks, *P* = 0.01). The author and Wilson et al. (2003) have the same conclusion that the repair of fetal myelomeningocele performed in pregnancy <23 weeks is related to premature rupture of membranes and separation of membranes.

Sydorak et al. (2002) carried out a retrospective study for CMS in 2022. They collected 75 samples of fetal surgery in their hospital within 10 years, checked whether there was CMS in their ultrasound images, and paid attention to their clinical prognosis. Excluding postoperative death, the incidence rate of CMS in all postoperative patients was 47%. There were significant differences (*P* < 0.05) between CMS and non-CMS in the time to delivery (7 weeks vs. 5 weeks), use of infusion pumps (80% vs. 60%) and the number of trocars (2.13 vs. 1.54). The incidence of premature rupture of membranes (63% vs. 45%), premature delivery (57% vs. 38%) and chorioamnionitis (29% vs. 15%) in CMS patients increased, but there was no difference in mortality. This study systematically reviewed the relationship between open fetal surgery and FM separation and showed that open FM surgery is an important cause of FM separation.

Ultrasound is mainly used for prenatal diagnosis in the first and second trimesters of pregnancy. Thin or thick membranes may be related to premature delivery, which is caused by a variety of physiological mechanisms leading to premature delivery. The occurrence of CMS is usually associated with poor fetal prognosis. As the most commonly used obstetric imaging technology, it is necessary to further study the characteristics of fetal membranes in ultrasound and poor clinical outcomes. However, there are still some problems to be solved in FM ultrasound imaging, such as small size, low resolution, the need for experienced doctors, subjective diagnosis, etc. (Basaran et al., 2014).

### FMs in MRI

MRI is not the best choice for prenatal imaging diagnosis; there are few studies on fetal membrane imaging using MRI, and only three related studies were retrieved.

Qi et al. (2020) used a new MRI method called three-dimensional constructive interference in steady state (3D-CISS) to detect FMs in the cervix region during pregnancy to explore FMs before rupture. The study included 18 pregnant women between gestational weeks 18 and 35 who underwent longitudinal MRI scans. The membranes of 14 women looked normal and intact, while the membranes of the other four women showed some abnormalities, including cervical funneling, CMS and chorionic rupture. They observed the gradual separation of the membranes of the four women at multiple time points. The results showed that, first, the FM stretched and protruded into the cervix when the cervical internal os dilated to cause cervical funneling; then, the amnion separated from the chorion, and the chorion ruptured. Finally, the amnion ruptured. This phenomenon is consistent with the conclusions of previous studies on *in vitro* models of FM rupture (Arikat et al., 2006). This is the first *in vivo* evidence of an *in vitro* model of the timing of rupture of membranes, PROM and pPROM. They believe that these findings may help to develop an *in vivo* MRI marker to improve the detection of FMs. However, this study has some limitations, for example, the sample size is small, other characteristics of the FM (thickness, etc.) are not measured, and pPROM is not separated from PTL in the follow-up record.

In 2021, Hutter et al. (2021) used MRI to observe the morphology of the placenta after premature rupture of membranes and performed MRI scanning of the placenta in 12 women before 34 weeks of gestation. The MRI images in this study can clearly show the characteristics of chorioamnionitis and FM structure, but unfortunately, they did not discuss the relationship between fetal membrane morphology and fetal prognosis.

Qi et al., 2022 used T2 weighted magnetic resonance imaging to observe FMs and used the observed indicators to predict PROM and pPROM. This prospective study cohort included 77 women between 28 and 37 weeks of gestation. They mainly studied two fetal membrane defect indicators, including FM prolapse depth >5 mm and abnormal signal. Fisher’s precision test was used to determine whether prolapse depth >5 mm and abnormal signals were related to PROM and pPROM. Then, they calculated the sensitivity, specificity, positive predictive value, negative predictive value and accuracy of prolapse depth > 5 mm, signal abnormality and the combination. Among the 12 PROM women (5 preterm and 7 term), 9 had FM prolapse >5 mm, and 5 had abnormal FM signals. Among 65 women with full-term rupture of membranes, 2 had rupture of membranes, and 1 had an abnormal FM signal. Fisher’s test showed that FM prolapse > 5 mm and FM signal abnormalities were correlated with PROM (*P* < 0.01) and pPROM (*P* < 0.01). The combination of the two indicators showed high specificity in predicting PROM (96.9%, 98.5% and 100%, respectively) and pPROM (90.3%, 97.2% and 100%, respectively). This study provided two FM MRI indicators to predict PROM and pPROM. In this study, MRI was used for the first time to predict PROM and pPROM in supracervical FM imaging.

MRI is a complementary examination of ultrasound, which can confirm the results of the ultrasound examination. However, for the theoretical risks of static magnetic field (B0), time-varying magnetic field and radio frequency (RF) pulse, radiologists should conduct risk benefit analysis of MRI examination (Mervak et al., 2019). Nonetheless, studying the characteristics of fetal membrane imaging in MRI can further assist the diagnosis of fetal membrane abnormalities in ultrasound.

### FMs in OCT

Medical imaging technologies such as ultrasound and MRI are widely used in prenatal diagnosis, but due to their insufficient spatial resolution and other technical defects (potential radiation hazards), they can only provide limited diagnostic value. Optical coherence tomography (OCT) is a new optical imaging method that distinguishes the organizational structure according to the absorption and reflection characteristics of light and produces high-definition and high-resolution real-time images (Men et al., 2016). If combined with endoscopy, it can improve the spatial resolution (1–10 μm), and the biological tissues can be imaged in 2D and 3D at medium depth (1–3 mm). In previous studies, OTC has been used to describe the histomorphology of the colon, bladder, and other tissues (Adler et al., 2009; Ren et al., 2010), and *in vivo* studies have proven that endoscopic OTC is clinically practical (Sergeev et al., 1997; Kang et al., 2011; Pahlevaninezhad et al., 2016). We found three studies used OTC methods to image FMs.

Ren et al. (2011) conducted a preliminary feasibility study on fresh human FMs from a control group and patients with microscopic chorionic pseudocysts (MCPs) to explore the potential of OCT for the early detection of pathological changes. They collected the FMs of 10 full-term cesarean-section pregnant women and took samples from different parts of the membranes (for example, near the anterior and posterior walls of the uterus, near the cervix). A total of 60 FM samples were collected. The results confirm that OTC can describe the morphological details of FMs into four layers (e.g., DV, CT, A, and E) according to their backscattering, with rCT/DV = 0.51 ± 0.17; rA/DV = 0.84 ± 0.45; RE/DV = 0.44 ± 0.20. Furthermore, MCP lesions (cysts) in the CT layer can be detected according to the significantly reduced backscattering of the membranes. More importantly, by postprocessing OTCs (segmentation and registration), OCT morphology detection may achieve quantitative and staging of MCP progression, which is crucial for monitoring the evaluation and treatment effect of preeclampsia. However, they also recognized that the imaging depth of OTC is limited, which may limit its diagnosis and evaluation of late severe MCP lesions. When there are a few specimens with thick DV layers (2–4 mm) on the maternal side, OTC cannot fully depict the layered structure of FMs. In this case, high-frequency ultrasound (HFU) can be used as a complementary method for OTC.

Micili et al. (2013) conducted a study on FM’s thickness using OTC. In this study, they collected 45 FMs after delivery, 36 of which were used for OTC measurement and 9 for histological measurement. According to the birth situation of the fetus, they divided the FMs into three groups: normal birth (NB), preterm birth (PRB) and pPROM. The study focused specifically on the rupture of the membranes, the middle of the membranes, and the margin of the placenta, and each group was analyzed independently. The OTC images were successfully obtained for all membranes, and the chorioamniotic structure was visible. The results showed that the membranes of the NB group were the thickest, the PRB group was the thinnest, the FM’s thickness of the pPROM group wasbetween the NB and PRB groups, and the membranes of the NB and pPROM groups were statistically significant.

Avila et al., 2014 collected FMs from 10 cesarean section patients (4 cases without pregnancy, 4 cases with preeclampsia, 2 cases with chorioamnionitis) and 8 vaginal delivery patients (6 cases without pregnancy, 2 cases with chorioamnionitis) and analyzed them with the OTC imaging method. The results illustrate that OTC clearly showed the structure of FMs, including the amnion, the subamniotic space, the reticular layer of the chorion, and the chorio-trophoblast-decidua. The amnion showed a bright band at the bottom of the OCT image. Other histopathological features identified by OCT include microscopic chorionic pseudocyst, ghost chorion, calcification, meconium staining, and chorioamnionitis. In this study, OTC imaging clearly showed the structure of the FM. Compared with the study of Ren et al. (2011), OTC imaging in this study shows a variety of pathological features corresponding to pathological sections. They believe that this feasibility study demonstrates the potential of OCT technology in the real-time evaluation of human FMs and may provide clinically useful information during childbirth.

OTC has made some achievements in the imaging of FMs, and it also has good performance in the display of FM structure and pathological characteristics. However, compared with OTC research in other diseases, there are still some limitations in the research of FM imaging (it is not possible to image a thicker FM and directly image it *in vivo*). However, with the improvement of OTC technology and the promotion of related research, the integration of OTC imaging with endoscopy holds potential as an effective diagnostic approach in prenatal diagnosis.

### Deep learning in future FMs imaging

Machine learning has played an important role in medical image processing. In the field of cancer, it has achieved good results in diagnosis and predicting treatment. Similarly, machine learning has great potential in fetal membrane imaging.

Firstly, object detection networks such as Yolo (Redmon et al., 2015) and Faster RCNN (Girshick et al., 2013) can achieve real-time object detection, while Unet (Ronneberger et al., 2015) and SegNet (Vijay et al., 2017) networks can achieve image segmentation. Both image detection and image segmentation algorithms can achieve automatic recognition of the fetal membrane. During fetal membrane examination, the position of the fetal membrane can be directly displayed, providing a more intuitive explanation of the diagnostic results. The fetal membrane includes the chorion, amnion, and decidua, which are composed of multiple layers of tissue. When it is necessary to specify the situation of a certain layer in diagnosis, machine learning can also be used to label different layers of the fetal membrane.

Based on deep convolutional neural networks and a large amount of fetal membrane data, machine learning can also judge the pathological state and possible adverse clinical outcomes of the fetal membrane. This classification technology is already quite mature, but requires a large amount of image data and clinical outcome labels as the basis for training.

Machine learning is playing an increasingly important role in modern medicine, as it not only enables rapid diagnosis to save human doctors’ time, but also solves the problem of a lack of experienced doctors in remote areas.

However, the training of machine learning is based on big data and is often difficult to explain, which requires more advanced algorithms to address the issues of data scarcity and interpretability. It is important to note that the successful implementation of deep learning in FM imaging requires the availability of large, well-curated datasets for training and validation purposes. Additionally, collaboration between medical professionals, imaging experts, and data scientists is essential to ensure the development of robust and clinically applicable deep learning models.

In conclusion, the application of deep learning techniques in future FM imaging holds immense promise, offering the potential for improved diagnostic accuracy, better understanding of FM pathologies, and enhanced patient care in obstetrics.

## Discussion

The abnormalities of FMs are closely related to fetal prognosis. The inflammatory response caused by chorioamnionitis may cause thickening of the fetal membrane (Severi et al., 2008), while fetal surgery and chromosomal abnormalities, could lead to the separation of the chorioamniotic membrane (Wilson et al., 2003; Soni et al., 2016; Gerson et al., 2019). In this review, the study of FM imaging *in vivo* is mainly conducted through ultrasound imaging. Various studies have yielded disparate findings regarding the association between membrane thickness and the occurrence of preterm birth. This may because of the mechanism of premature rupture of membranes caused by inflammation, infection and other complicated factors (Nunes et al., 2016). This could also because the position or time of the FM measurement is inconsistent, which leads to deviation in the observation results. The CMS is related to the premature delivery or the fetus, which is related not only to inflammation and infection but also to chromosome defects (Gerson et al., 2019). In MRI imaging, a paper discussed the gradual change process before FM rupture using the MRI method, which is consistent with the previous research conclusions of some *in vitro* models of FM rupture. Another paper discussed that FM prolapse and FM signal abnormalities, were associated with PROM and pPROM.

For *in vitro* imaging of FMs, the OCT method is mainly used. OTC has made some achievements in the imaging of FMs, and it also has good performance in the display of FM structure and pathological characteristics. However, compared with OTC research in other diseases, there are still some limitations in FM imaging research (it is impossible to image a thicker FM and directly image it *in vivo*) (Ren et al., 2011).

The majority of these studies have been limited by small sample sizes, thereby restricting their applicability as definitive guidelines for clinical diagnosis and treatment. To obtain more substantial and reliable outcomes, future endeavors should focus on conducting large-scale imaging research initiatives. Such endeavors hold the potential to yield more meaningful and robust results with enhanced clinical relevance.

In addition to the fetal membrane imaging of ultrasound, MRI and OCT mentioned in the article, some imaging technologies have the potential for fetal membrane imaging. These include optical coherence elastography (OCE) (Pitre et al., 2020; Bhunia et al., 2022), magnetic resonance elastography (MRE) (Liang et al., 2008; Jiang et al., 2014), and shear wave elastography (SWE) (Li et al., 2012; Hernandez-Andrade et al., 2014). However, these methods are still relatively new, and the research is not perfect, so we need to further explore their feasibility in fetal membrane imaging.

Moreover, with the development of artificial intelligence (AI) and deep learning (DL), more and more researchers begin to apply AI models to medical images. AI has developed diagnostic models with clinical significance based on medical images for tumors (Li et al., 2019; Zhou et al., 2020; Murakami et al., 2021), eye diseases (Milea et al., 2020; Murakami et al., 2021), cardiovascular diseases (Dey et al., 2019; Fernandez-Ruiz, 2019) and other diseases. However, in the field of fetal membrane imaging, the research on artificial intelligence is still blank. AI can play a variety of roles in medical images. First, it can segment and recognize medical images. In tumor images, the segmentation of tumors and nodules has been quite mature (Gietema et al., 2007; Wang et al., 2017; Zhang et al., 2022). In the field of obstetrics and gynecology, there are also studies on MRI placental segmentation (Pietsch et al., 2021; Gaga, 2022). Such technology can also segment the fetal membrane region; Secondly, based on image segmentation, AI can achieve automatic measurement, such as NT value measurement and nodule size measurement (Abele et al., 2010; Bakker et al., 2013). Similarly, such measurement can also be applied to the measurement of fetal membrane thickness; Finally, the image-based diagnosis model is also an important direction of AI in fetal membrane imaging, but the image AI diagnosis model of fetal membrane requires a large number of image data as the research basis.

According to previous studies, fetal membrane abnormalities are closely related to adverse pregnancy outcomes. Using AI can learn the features of FM images, to help human doctors make a fast and accurate diagnosis, and also solve the problem of lacking experienced imaging doctors in remote areas. Finally, for large-scale cohort learning, AI can reduce the subjectivity of human doctors’ diagnosis, thus ensuring the objectivity of research. AI has a broad prospect in the research of FM imaging.

## Conclusion

Studying fetal membrane imaging is of great significance for the diagnosis of pregnancy complications and fetal malformations. *In vivo* research is mainly focused on ultrasound, and a small number of studies use MRI to study FMs. *In vitro* imaging is mainly focused on OCT. However, it is noteworthy that the majority of these studies suffer from limited sample sizes and exhibit inconsistent conclusions. Moving forward, a comprehensive assessment of FMs through the integration of multiple imaging techniques may yield a more comprehensive depiction of their state. Furthermore, amalgamating these findings with extensive large-scale data research can generate conclusions of heightened clinical relevance. Leveraging machine learning approaches to analyze FM images can further aid clinicians in diagnosis, encompassing the prediction of premature birth and other adverse clinical outcomes via medical imaging. This innovation bears the potential to address challenges related to the scarcity of senior physicians and a dearth of specialized healthcare professionals in remote areas.
